# 1889

**Published:** 1889-01

**Authors:** 


### EDITORIAL.

### 1889.

The year 1888 has gone into the past and the work of that year is irrevocably fixed, historically at least. For all the good that was done may all be sincerely thankful, and resolve that in this respect the present year shall have a better record than the past.

And while naught but regret can be entertained for the wrong-doing, yet it may be used as a beacon, warning us of dangers upon which so many have run and suffered, and upon which multitudes have been lost.

Let us increase the good and diminish the wrong—stand for the right and inflexibly against the wrong.

In the practice of our profession, and in all that we do for it, may our aim be onward and upward; let our efforts be to ascertain and establish so far as may be, the true value of many things that have been introduced but not yet fully proven.

Each successive year shows greater progress than any preceding one, and with this advance comes increasing responsibility. This pertains to all departments of our professional work—association, journalistic and educational.

Let each one see to it, that he adds something to the progress of 1889. It is better that each and every one should help a little than that any *one* should make a great stride.

The practice of American dentists abroad, especially in European countries, has become an interesting one. There is in almost every country in Europe legislative enactments regulating the practice of dentistry, and this not only for the natives who practice in the respective countries, but especially for foreigners.

Fifteen years ago, dentists going from the United States to almost any country of Europe had free scope to exercise his vocation, at least so far as law was concerned. But it is now far otherwise. The question naturally arises why this change? Years ago only the better qualified and successful dentists sought fields abroad, and they usually attained success, both as to establishing a reputation, and financially.

More recently, however, in addition to some men of high attainments, many of inferior ability, and not a few lacking integrity as well, have gone abroad and in too many instances have by charlatanism brought reproach and disgrace, not only upon themselves but in a measure upon the profession in this country. As bearing upon, and evidence in this matter the following quotation from the *British Journal of Dental Science*, Oct. 15th, is pertinent:

"AN AMERICAN JOURNALIST ON AMERICAN DENTISTS.—The trouble with the American dentists who were fined in London courts recently was too much unprofessional publicity. A score of other American dentists here, who went about their work quietly, were not disturbed, though, now that a test case has resulted in favor of the Britishers, the probability is that they will be, unless they find some technical means of evading the law. Only dental graduates from Harvard and Michigan schools are recognized by the British Dental Association. (sic!) If a young dentist from the New York College, or a dentist from any other dental institution except the two mentioned, comes over here to practice, he must take a two years' course. A prominent London dentist said to-day: 'This is not persecution of good American dentists, for capable men will not be interfered with; but the average American dentist in London would astonish the Americans as much as the average American bar.' We quote the above with a purpose, 'American papers please copy.' We cannot be so sure of the safety of 'good American dentists,' for law-breakers, however good, are apt to become entangled by the web of the law. An American journal, which by the way ought to know better, as it is run under the names of well-known and respectable men, has accused us of American-dentist-phobia, and we beg to bow our heads to the impeachment. When our critic comes across the briny, we shall be pleased to show him a few samples of the 'American dentists,' towards whom we are 'phobic,' and if he wishes to extend his brotherly arms to these gentry, why let him, only we shall cease to exchange. For honorable straightforward Americans practicing dentistry, and who scorn hanky-panky, we entertain a high regard, and candid readers of the *British Journal of Dental Science* know that fact so well that we need say no more."

